# Biodiversity information platforms: From standards to interoperability

**DOI:** 10.3897/zookeys.150.2166

**Published:** 2011-11-28

**Authors:** W. G. Berendsohn, A. Güntsch, N. Hoffmann, A. Kohlbecker, K. Luther, A. Müller

**Affiliations:** 1 Department of Biodiversity Informatics and Laboratories, Botanic Garden and Botanical Museum Berlin-Dahlem, Freie Universität Berlin, Königin-Luise-Straße 6-8, 14195 Berlin, Germany

**Keywords:** EDIT, Common Data Model, CDM, Scratchpads, Standards, TDWG, biowikifarm, Taxonomy, Biodiversity, Biodiversity informatics

## Abstract

One of the most serious bottlenecks in the scientific workflows of biodiversity sciences is the need to integrate data from different sources, software applications, and services for analysis, visualisation and publication. For more than a quarter of a century the TDWG Biodiversity Information Standards organisation has a central role in defining and promoting data standards and protocols supporting interoperability between disparate and locally distributed systems.Although often not sufficiently recognized, TDWG standards are the foundation of many popular Biodiversity Informatics applications and infrastructures ranging from small desktop software solutions to large scale international data networks. However, individual scientists and groups of collaborating scientist have difficulties in fully exploiting the potential of standards that are often notoriously complex, lack non-technical documentations, and use different representations and underlying technologies. In the last few years, a series of initiatives such as Scratchpads, the EDIT Platform for Cybertaxonomy, and biowikifarm have started to implement and set up virtual work platforms for biodiversity sciences which shield their users from the complexity of the underlying standards. Apart from being practical work-horses for numerous working processes related to biodiversity sciences, they can be seen as information brokers mediating information between multiple data standards and protocols.The ViBRANT project will further strengthen the flexibility and power of virtual biodiversity working platforms by building software interfaces between them, thus facilitating essential information flows needed for comprehensive data exchange, data indexing, web-publication, and versioning. This work will make an important contribution to the shaping of an international, interoperable, and user-oriented biodiversity information infrastructure.

## Introduction

In the last two to three decades there was a growing recognition that biological diversity is a global asset of tremendous value to present and future generations (Convention on Biological Diversity, UN 1992). This has led to a rising number of projects that gather data in the domain of biodiversity. The central component of biodiversity is organismic diversity, which is largely described in terms of systematics (the classification of organisms into taxonomic groups such as species), biogeography (the geographical distribution of the taxa in past and present), and synecology (the interaction of organisms in communities). “Biodiversity informatics” (Anon. 1999) focuses on this level of biodiversity, whilst the closely related and interacting of ecoinformatics and bioinformatics concentrate on ecosystems and on the molecular and related physiological level, respectively. Biodiversity informatics addresses data from preserved collections (natural history museums, herbaria), living collections (botanical and zoological gardens and culture collections), as well as from data collections from research (e.g. floristic and faunistic mapping, monitoring) and citizen science initiatives (e.g. bird watching). Another important data source is literature, especially taxonomic literature, which in its entirety (going back for more than 250 years) continues to be highly relevant for today’s research. Last but not least, output from on-going research in systematics and synecology provides an ever-growing amount of data, extending into diverse new data types like cladograms, multimedia records of species, the specific data needed for new types of collections (e.g. DNA banks, Gemeinholzer et al. 2011), and a growing body of evidence about important functional attributes of organisms, such as a multitude of ecological traits, and also their potential to be invasive or serve as a vector for diseases.

Efforts to share these data soon led to the realisation that capture and storage of biodiversity data is not enough; although most of the attributes are shared across the entire domain, the data sets are not easily linked or integrated. The lack of shared vocabularies and the diversity of data structures used has impeded (and still impedes) the sharing of data. Data sharing is essential to facilitate the collaboration and large-scale analysis needed for a successful treatment of the pressing issues connected with biodiversity. Standards provide a consistent representation of the data to be shared enabling data from different sources to be combined, whilst minimising loss or duplication of data.

“Biodiversity Information Standards (TDWG)” is an organisation that works on defining such standards in the field of biodiversity informatics. TDWG was originally established as the “Taxonomic Databases Working Group” by major botanical institutions and projects from around the globe in 1985 (Anon. 2007). Task groups within TDWG initially worked on data dictionaries and exchange standards for botanical databases. Early examples for exchange standards are the “International Transfer Format for Botanical Garden Data” (ITF, IUCN/WWF 1987) and the “Herbarium Information Standard and Protocol for Interchange of Data“ (HISPID, Croft 1992). The “Descriptive Language for Taxonomy“ (DELTA, Dallwitz 1980) was accepted for the encoding of taxonomic descriptions and identification keys. Standard works listing abbreviations for periodicals (Bridson and Smith 1991) and taxon authors (Brummitt and Powell 1992) as well as a newly devised standard scheme for geographical areas (Hollis and Brummitt 1992) were accepted as data standards. In the 1990s, the focus shifted to work on data models, which in turn revealed the high complexity of the domain (e.g. Berendsohn and Nimis 2000). Modelling efforts, albeit sometimes leading to extensive discussions of minute details, did serve to further stabilise the usage of terms and data format definitions in the domain (see Berendsohn 2005 for a compilation). The scope of TDWG was widened to include all organism groups and reached out beyond the taxonomic community, which recently also led to changing the organisation’s name to “Biodiversity Information Standards (TDWG)”. In the last decade, much discussion centred on community protocols for data exchange on the Internet, and the definition of appropriate XML schemas for data exchange. Based on all these developments, the discussion of how to achieve a joint semantic and structural description for domain specific data was recently revived (now under the term “ontology”) and also included in the workplan of the ViBRANT project.

To be able to discuss the role of Biodiversity Information Platforms we need to have an exemplary look at some of the TDWG standards and other formats currently used in the field of taxonomy.

ABCD (Access to Biological Collection Data) and DwC (Darwin Core) are two standards intended to support the exchange of collection and observation data. Both have been ratified by TDWG as standard XML schemas. The ABCD standard (see Berendsohn 2007) set out to capture all data elements used in specimen and observation data collections that may be provided by collection information systems. It comprises nearly 1200 elements and attributes (including several hundred which are descriptors of elements, e.g. for language). No collection uses more than a fraction of the elements defined in ABCD, but the set of elements used varies greatly. The ABCD standard is directly used by the Global Biodiversity Information Facility (GBIF) and the Biological Collection Access Service (BioCASe). It has been extended to support the DNA Bank Network, the GeoCASE portal (“ABCDEFG”, the “extension for geosciences”) and the latest version of HISPID.

The DwC standard (Wieczorek et al. 2009) describes the occurrence of species and the existence of specimens in collections. It is a smaller set of data element definitions also designed to support the sharing and integration of primary biodiversity data. Efforts were made to keep DwC and ABCD largely compatible on the element level. DwC draft 1.4 is under discussion but already used in GBIF. Version 1.2 is used e.g. in the MaNIS (Mammal Networked Information System) and ORNIS projects (Stein and Wieczorek 2004). A variety of DwC is also used in the Ocean Biogeographic Information System (OBIS, Halpin et al. 2009).

TCS (Taxonomic Concept Transfer Schema, Anon. 2005) was developed for exchanging taxonomic data. However, TCS defines only the structure of the taxonomic backbone. For a broader export/import of taxonomic data other formats have to be used in addition (e.g. ABCD or DwC).

SDD (Structured Descriptive Data, Hagedorn et al. 2005) is the current TDWG standard for descriptive data. Many of the existing descriptive data managing tools, e.g. Lucid (Anon. 2010), Xper² (Ung et al. 2010), and DiversityDescriptions (Weiss et al. 2008) already support import and export of SDD conformant data, allowing their users to exchange highly structured descriptive data. See Hagedorn (2007) for references and an in-depth analysis of descriptive data and tools.

DwC-A (Darwin Core Archives, Robertson et al. 2009) is an updated and extended version of DwC. It is developed by GBIF in the context of the Global Names Architecture (GNA, Anon. 2011). DwC-A is based on the DwC terms and the DwC text guidelines, however, the extended version is not limited to occurrence data but also covers organism names, taxonomies, species information, factual data, distributions, media, and literature.

Taxonomy relies on the results of more than 250 years of research laid down in scientific publications. Digitisation of this content is well under way, but to become truly useful the content needs to be converted into structured databases. Efforts are under way to standardise the markup for the content of taxonomic literature as a prerequisite for this process. TaxPub is an extension of NLM/NCBI Journal Publishing DTD (Version 3.0) that adds elements and attributes relevant to taxonomic descriptions to the already included elements for document features (Catapano 2010). From within the community, the TaxonX schema (Sautter et al. 2009) was developed to streamline the process of text markup. See also Penev et al. (2011) for further information.

The work done has led to a comprehensive overview of the data in the highly complex domain of biodiversity informatics. But all these modelling efforts and resulting standards have no effect if the applications the researchers use cannot import and export standardised data. This is only starting to happen. For example, tools for descriptive data can exchange data using SDD, and some formats that are not (yet) TDWG standards such as Species Profile (Anon. 2009) and Plinian Core (Anon. 2007) are in practical use for data sharing by a number of applications (LifeDesks, Scratchpads, content partners of the Encyclopedia of Life and a variety of Spanish-language tools).

There is a need for workflow-based approaches for converting and integrating data and shielding the user from the complexity of the standards and data structures. Focusing on this problem, the European Distributed Institute of Taxonomy (EDIT) created the EDIT Platform for Cybertaxonomy. The EDIT Platform supports the entire taxonomic workflow, therefore it provides possibilities to import and export data in a standardised way (ABCD, DwC, SDD). Additionally, the EDIT-funded Scratchpads provide a scalable data publishing framework with flexible data models that can be modified by its users.

## Biodiversity information platforms as information brokers

Lack of interoperability is one of the major obstacles to establishment of efficient workflows that help scientists and other users and user groups of the Biodiversity Informatics Infrastructures to improve quality and efficiency of their working processes. Advanced workflow management systems such as Taverna (Hull et al. 2006) and Kepler (Altintas et al. 2004) can greatly improve the execution of service-driven processes. However, there are still considerable technical barriers to overcome for users who wish to compose or re-use workflows from disparate services and data standards. Moreover, workflow management systems do not attempt to be comprehensive and to provide a complete working environment for entire research areas. Rather, they offer the means to streamline very specific sequences of data operations, which are time consuming and occur often in the day-to-day work processes.

In contrast, the emerging biodiversity information platforms implement a different and complementary approach by trying to cover many different scientific and other working activities and hiding underlying data models and access protocols completely from their users. These platforms are usually centred around a local or distributed data store based on a comprehensive information model providing a unified instance of all data needed for scientific activities ranging from field work to data publication on paper and in web portals. Moreover, biodiversity information platforms provide the necessary interfaces to deploy external software tools and services in a way that users can still work with often highly specialised software applications they are used to. Data from external applications can be seamlessly integrated and further processed in the platform environment. In this way, biodiversity information platforms exploit their potential as information brokers and help users to benefit from information standards, which they would be unable to deploy otherwise.

The ViBRANT project work package 4 (standardisation) aims to improve interoperability between biodiversity information platforms and focuses on three emerging systems: Scratchpads (Anon. 2006), EDIT Platform for Cybertaxonomy (Anon. 2007), and biowikifarm (Metawiki contributors 2011), which are briefly outlined in this section.

### Scratchpads

The software platform Scratchpads (Smith et al. 2009) has been initiated by the European Distributed Institute of Taxonomy (EDIT) and is based at the Natural History Museum in London. The key aim of Scratchpads is to provide a scalable data publishing framework with flexible data models that can be modified or added to by its users. Automated integration of third party content and automated semantic enrichment of contributed and third party content are further key features of this platform. The principle design decisions for this platform are founded on the insight that the effort (transaction cost) required by users to sufficiently structure (or restructure) their data is too high, relative to their perceived benefit from using the system. Thus it provides users with a system that allows assembling data quickly in a semi structured way.

Scratchpads are build on the content management system Drupal (2001), originally using version 5; at the time of writing it is being transitioned to Drupal 7. Making use of the data structuring principles provided by Drupal, data is organised around term vocabularies, such as biological classifications of taxon names. These vocabularies can be associated with various content types. Content is managed in so-called nodes, which can contain structured or semi structured data depending on the given content type. Structured content types are provided by specific modules like the biblio module (Jerome 2006) that allows users to manage and display lists of scholarly publications. The character node type allows users to build and manage structured descriptions of taxa in a controlled matrix. The set of predefined content types can be complemented by custom content types which users can define to adapt their scratchpad to their needs. This approach provides flexibility to accommodate use cases that were not originally envisaged, but at the cost of heterogenic data structures between the various scratchpad instances.

The content entities are related to each other by tagging them with terms from the associated vocabularies. In that sense taxonomic names provide a central link between diverse items of information about a taxon. Scratchpad taxon pages allow users to dynamically construct and curate pages of information (e.g. phenotypic, genomic, images, specimens, geographic distribution). External data from some third party data services (bhl, flickr, wikipedia, yahooimages) can also be dynamically aggregated into these taxon pages. Data provided by web services, however, in general can be placed only into taxon pages; it is not possible to integrate and process them in the local data structure. The only exception is taxonomic classifications which can be obtained in form of uBio ClassificationBank for Species 2000, ITIS and NCBI Genbank.

File based imports exists for classification terms, locations and specimen data which are all based on the CSV (Comma-Separated Values) file format. Following the principle of high flexibility none of these imports requires the data fields to be ordered in a predefined structure, thus these imports always involve user interaction and cannot be automated. Structured data in standardised data exchange formats only exist for bibliographic data. The Scratchpads can import Tagged EndNote, RIS, MARC, EndNote 7 XML, EndNote 8+ XML and BibTeX formatted bibliographies.

Scratchpads provide a limited range of services to expose data to other software systems. At present these are restricted to specimen and bibliographic data (Smith et al. 2009). Specimen data is provided by TapirLink software (DeGiovanni et al. 2007 external to the Scratchpads. TapirLink uses each set of Scratchpad database specimen records as a data source. These data fit the DarwinCore v1.2.1 standard (Wieczorek and al. 2009). Bibliographic data are currently available from the Scratchpads in BibTeX or Endnote format. Bibliographic data is also exposed using the OAI-PMH (Open Archives Initiative Protocol for Metadata Harvesting) module (OAI undated). In addition, Scratchpad users can create views of their data in arbitrary XML formats that can be accessed by others. There is a special module to export descriptive data to EOL (C. Parr, in litt.).

The flexibility which allows adapting scratchpads to individual needs leads to semi-structured heterogenic data, which often hinders their integration into service-oriented software environments. A major task in achieving better platform interoperability will be to implement web service APIs, which communicate data in commonly accepted exchange formats.

### EDIT Platform for Cybertaxonomy

The EDIT Platform for Cybertaxonomy (Berendsohn 2010), henceforth called “EDIT Platform” provides researchers with a set of coupled tools for: full, customised access to taxonomic data; editing and management of data; collaborative work in teams; and efficient publishing to both the web and in printed form. The EDIT Platform has been funded through the EDIT (European Distributed Institute of Taxonomy) project. Development of the EDIT Platform is coordinated by the Dept. of Biodiversity Informatics at the Botanic Garden and Botanical Museum Berlin-Dahlem, and its various components are being evolved by a team of software developers and architects from institutions all over Europe.

Establishing interoperability between various existing applications and data standards was a major aim in developing the EDIT Platform. A central data repository and information broker application has been created to achieve interoperability with and between existing applications and web based data providers. It allows other software to exchange data, via import and export functionality in major data formats, or via web services.

This data repository as well as the core components “EDIT Taxonomic Editor” and “EDIT DataPortal” are based on the EDIT Common Data Model (CDM), which comprehensively covers the information domain, including nomenclature, taxonomy, descriptive data, media, geographic information, literature, specimens, and persons. Wherever possible, the CDM has been made compatible with existing community data standards. This model as a base allows managing data consistently in highly structured form. A Java (Oracle 2011) application programming interface (API) for the CDM makes it easy to develop new CDM applications and to integrate existing applications. An example for the latter is the integration of Xper² (Ung et al. 2010) with the CDM. The CDM library provides an import and export package for taxonomic classifications, descriptive data, specimens and observations, and media in many standardised or quasi standardised data formats such as SDD, DarwinCore, TCS/RDF, TCS/XML, TaxonX and several MS Excel formats especially developed and in use for biodiversity data. In addition to that a generic XML export exists which allows dumping the entire CDM data base into a file and reimporting it.

The import and export functionalities are complemented by web services exposed by the CDM Community Server (EDIT 2011), a standalone server application which can be connected to any CDM database. The major web service is the CDM REST (representational state transfer) API (EDIT 2011a), a RESTful (Fielding 2000) interface to all resources stored in the CDM. This web service exposes data items as XML or JSON serialisations. For example the EDIT DataPortal extensively uses the access points provided by this generic web service API; the same is true for the print publisher tool built into the EDIT Taxonomic Editor software. In perspective, this web service API is an excellent base for the future integration of EDIT Platform functionality into the above mentioned workflow environments; it needs only minor extensions in order to fully conform to these environments.

Another web service implements the OAI-PMH (Open Archives Initiative Protocol for Metadata Harvesting, OAI undated) specification and thus allows aggregators like the Biodiversity Heritage Library (BHL), GBIF, and the Encyclopedia of Life (EOL) to harvest reference and taxon items selectively. However, for such large-scale aggregators who wish to harvest entire datasets and keep indices local and fresh, Darwin Core Archive is a better option.

EDIT Platform components like the EDIT Taxonomic Editor can directly use external data providers. This is made possible by service wrappers allowing querying, retrieving and integrating of external data into a CDM data store, where these remote objects can be reused, without losing the information on their origin. Service wrappers already exist for specific data providers like the International Plant Names Index (IPNI 2008) and the Biodiversity Collections Index (BCI 2007. Other services implement widespread web service search protocols like OpenURL (OCLC 2003 and SRU (Search/Retrieve via URL, Library of Congress 2011) and thus enable all CDM based EDIT Platform components to find and integrate data from any data provider which exposes its data through these search interfaces.

The EDIT Platform architecture is mainly service oriented, thus all data flows between different applications are established through web services. The EDIT Map Services, for example, produce distribution and occurrence maps based on data coming from a CDM Store. The communication between both components is effected through a URI based web service API.

**Figure 1.**
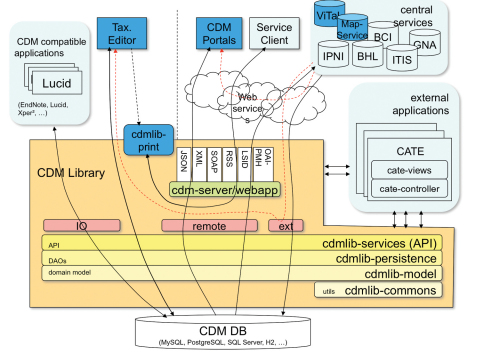
Architecture of the CDM Library and the EDIT Platform

### biowikifarm

Biowikifarm.net (Hagedorn et al. 2010) is a shared technical platform supporting a number of MediaWiki installations used by a diverse array of projects in biology, and especially in biodiversity research. The primary purpose of the shared platform is to enable long-term maintenance of the published data and to work more efficiently by distributing administrative and maintenance work among several partners. Furthermore, the biowikifarm operates a shared media repository, enabling synergies in re-using media content.

Using MediaWiki as technological basis allows biowikifarm to focus on the “long tail” of scientific information (Heydorn 2008). Supporting integration and preservation of this specific kind of unstructured or low structured data is a key feature by which it distinguishes itself from Scratchpads and the EDIT platform.

The biowikifarm is part of the activities of Plazi (Anon. 2008. It is maintained through the Julius Kühn Institute (JKI; programming and management) and the Botanic Garden and Botanical Museum Berlin-Dahlem (BGBM; technical support and hosting). The IT Centre of the Bavarian Natural History Collections (SNSB) is guaranteeing long-term online availability should dedicated project funds run out. In addition, users of the biowikifarm provide a significant contribution to the management of the farm.

Through the MediaWiki API, the software can be used as a service-oriented architecture, providing services to obtain page, file and relation objects (links, categories, semantic properties, data records) in a wide variety of data formats, including xml, rdf, json, html, and plain text.

Each of the MediaWiki installations contributing to this shared repository has different data structures, from simple arbitrarily structured wiki text pages to wikis like the “Offene Naturführer” (Anon. 2011a), which collects nature handbooks and determination guides in semi structured wiki pages. Even if all of them are sharing a common web service API the heterogenic and often unstructured nature of the content makes it hard to integrate the biowikifarm into workflow environments. This is a task, which has to be accomplished for each partner’s MediaWiki individually.

## Bringing it all together

None of the described platforms existed 5 years ago nor was there any commonly used tool available to edit and share biodiversity data in general and taxonomic core data in particular. At that time most applications designed for explicit handling of taxonomic core data (names, concepts, classifications) were in-house products, covering only the restricted requirements of local users and not supporting import and export of data in any standard format, perhaps with the sole exception of the databases providing collection and observation data in GBIF and related networks. User driven export of data to share it with other applications or projects was either not possible or ended up in user defined formats. An example is provided by the Global Compositae Checklist (Flann 2009, a project that aims to build up a global Compositae checklist based on local checklists. According to the coordinator (C. Flann, pers. comm.) they had to digest 55 different formats for a total of 67 data sets coming from 57 different sources, with only 1 dataset fully compliant with TCS the official standard for taxon classification data (several more were at least using TCS data definitions).

Obviously there was a considerable need for applications providing a joint platform for such projects. However, building them is more challenging then generally expected. To mention only the main obstacles: (1) a very complex and broad data domain covering the major fields of taxonomy and nomenclature, specimen and observations, descriptive data, literature, media, molecular data, and more; (2) high demands on the usability of user interfaces which may cover all the complexity of the domain; (3) the absence of a standard that covers all the domain – existing standards cover only parts and often overlap; and (4) the huge number of use cases to cover. The development is further complicated by the prerequisites for sustainable interoperability, namely (5) the demand for a generic open architecture that allows users with IT skills to adapt the software to their needs and allows participation in development (open source approach), (6) the demand for independence from hardware and operating system and database management platforms, and (7) the demand for web services, which make data and functions machine accessible and thus allow integration with other applications and with automated workflows.

Over the past years the described platforms first concentrated on the implementation of their core functionality, enabling users to do their every-day work of compiling, editing and integrating data, and publish the results on the web or as a print publication. At the same time, the basis for more advanced features was laid by building the systems using flexible and generic (though very different) architectures, as described in the previous section.

At present, all platforms have left the prototype status and are used to create content of high value. Although the list of demanded improvements and additional features is still long, it is now time to take a step back and reconsider how to integrate this content into the larger biodiversity e-infrastructure and how to connect the platforms in a way that creates additional value. All three platforms as well as other platforms like the emerging GBIF checklist bank (GBIF 2010) have specific characteristics that make them attractive for certain users and certain use cases. For some of these use cases one may want to transfer data from one platform to another either manually or in an automated way using web service infrastructures.

As an example, we want to use the capability of the EDIT Platform to act as a data warehouse handling multiple classifications within one database for complex high level queries on several datasets compiled using Scratchpads and CDM implementations. For this, periodic import of all relevant data of the respective Scratchpads and CDM Data Stores into a CDM based database will be needed. An automated procedure using a service producing DwC Archive as the transfer format is being devised for this purpose. It is also envisioned to use the result as a contribution to the Global Names Architecture, once its setup becomes clear.

There are a number of other use cases for data exchange between platforms. Single users or user groups may want to compile data within one system but synchronise them with a repository based on another system to use it for other reasons. This use case is comparable with the handling of contact data. Present-day users do not necessarily hold their contact data within only one system but synchronise them among systems and tools each of them having their own purpose (direct calls, exchanging v-cards, sending FAXes, creating serial letters, advanced backup, synchronisation, etc.). Also with emerging requirements and growing software functionality users may want to switch systems without losing data or having to re-enter it manually, just as they may change their preferred mail client or word processing tool from time to time.

Moreover, other platforms can be used for backing up or versioning data. This may be a preferred use of biowikifarm, taking advantage of the highly developed versioning technology of MediaWiki. Exporting data from one platform to a MediaWiki may serve as a perfect way to fulfil the requirement of providing stable and accessible versions within a constantly changing data environment.

As described in the sections above, the Scratchpads as well as the EDIT Platform do already support a number of available and commonly used standards for data exchange. However, as most existing TDWG standards and other commonly used formats handle only a subset of the full data domain managed by the platforms these standards have only limited value for inter-platform data exchange. They are preferably to be used for the initial import or to enrich existing data. For example, ABCD and Darwin Core imports are used to add specimen data to the existing taxonomy data. SDD can be used to enrich taxon records by supplementing them with highly structured descriptive data. Also the various literature formats are very helpful for enriching a community site with a commonly shared literature repository. Users of platform software can communicate with, for example, the Biodiversity Heritage Library, both to use the indexed and digitised taxonomic literature, and to provide information on missing titles. On the export side existing standards like ABCD and Darwin Core are used to expose data subsets like specimen data to data aggregators like GBIF by using existing wrapper technologies such as BioCASe (Holetschek 2005 or TapirLink (DeGiovanni et al. 2007).

However, most of the standards or standard implementations have drawbacks as they cover only a certain slice of the data, or are not widely accepted by the community, and/or are not yet fully implemented or are implemented only for either import or export, not both. Also, many of the implementations are file-based, and do not provide the web services needed for use within automated workflows. This may be circumvented by implementing further software similar to the BioCASE software that is used for web service data exchange of ABCD data.

An interesting question in the context of web services is the problem of data rights and licenses. Where the schema for the resulting document does not contain a space for licensing information, presently there is no way for a user (especially a machine) to discern the licensing terms under which the data is provided. Care has thus to be taken to include this information, where possible (e.g. in the metadata for Darwin Core Archive or in the metadata section of the ABCD schema). Where not, appropriate service extensions must be defined. Of course, in an ideal world the data would be provided under a Creative Commons 0 license (see Hagedorn et al. 2011), with no rights reserved; but even that has to be made explicit.

For taxonomy-centred datasets TCS - the official TDWG standard for exchanging taxonomic data - should be the preferred format. However, TCS defines only the structure of the taxonomic backbone. Other data types such as specimen, literature or descriptive data need to be explicitly implemented using another format. This causes problems when trying to exchange broad and rich data like those stored in the Scratchpads or the EDIT Platform. Both sides need to support not only TCS but also all extensions used by the other side to fill in the gaps. As this requires considerable coordination efforts TCS export has not yet been fully implemented by both platforms and TCS imports still have limitations.

Darwin Core Archive (DwC-A), a new format developed by GBIF and others tries to address the described problems by offering a more comprehensive data format that covers all major areas of biodiversity data. It currently comes in two different flavours either taxonomy centred or specimen centred – but other implementations are possible. Although DwC-A has its limitations it is already much more widely used than TCS due to its ease of use and relative unambiguousness. However, all three platforms currently support DwC-A only in parts or not at all. Within ViBRANT this will be addressed. DwC-A import and export functionality will be implemented for the Scratchpads and the EDIT Platform; for MediaWiki it is probably sufficient to implement import functionality.

As DwC-A is primarily a file based exchange format a harvesting mechanism will be implemented to complement the export functionality, which allows automated harvesting of DwC-A data via web services. Within ViBRANT, this technology will be used in particular to integrate all Scratchpad data within one large EDIT Platform based database to allow visualisation and advanced querying across-Scratchpad (and EDIT Platform) data. The service implementation will enable users to provide access to selected slices of the dataset (e.g. to exclude access to data on research in progress). Adequate filter mechanisms need to be implemented that allow definition of exactly which data should be exported. This may become a challenging task due to the complexity of the respective data models.

In contrast to the Scratchpads and especially the EDIT Platform, the data in MediaWikis are often unstructured or at most semi-structured. Creating generic export functionality for them will thus be very difficult, involving potentially extensive data curation measures, rendering it impractical in most cases. However, importing data will be straightforward and will enable users to use this platform as a repository for versioning and publishing. DwC-A could be used here as an exchange format, but as MediaWiki is a mainly text based system it may be better suited to export formats used for text publications. For example the emerging publication format TaxPub (Anon. 2008a maybe more appropriate for this purpose. Within ViBRANT the format best suited for exporting data to MediaWiki will be investigated and a data flow based on the selected format will be implemented.

## Conclusion

Biodiversity informatics faces an increasing need for integrated working environments facilitating efficient and streamlined data capture, processing, and publishing based on community standards. The EDIT Platform for Cybertaxonomy, Scratchpads, and biowikifarm each provide practical innovative software solutions which help their users who wish to organise their data in a standardised and networked manner. Further integration will be achieved in the course of the ViBRANT project by designing and implementing interfaces between the technologies. In work package 4 (“Standardisation”), the development of several data exchange modules will contribute to an improved overall interoperability between the EDIT Platform, Scratchpads and the biowikifarm as well as facilitate external connectivity. Based on a new DwC-A export module for Scratchpads and a corresponding import function built into the Java-API of the EDIT Platform for Cybertaxonomy, a comprehensive data index across all Scratchpad and CDM Datastore instances can be realised for the first time. The index will serve both (human) users wishing to perform cross-platform searches and software systems that need machine readable access to the “ViBRANT universe”.

Connectivity between CDM stores and Scratchpads as well as CDM stores and the biowikifarm platform will be realised with XSL transformation of CDM XML publishing output. Based on these pipelines, data managed by an EDIT Platform installation can be further processed in a Scratchpad. Stable versions can be created with exports into the biowiki platform providing a semi-structured and addressable snapshot of dynamic taxonomic databases.

The processing of descriptive data will be handled as a complementary mechanism using the Xper² system. For this, SDD-based interfaces between the EDIT-Platform and Xper² will be implemented and optimised for the transfer of high volumes of descriptive information. Collaborative compilation and development of new character- and character state lists will be enabled through the biowikifarm system. A service for the generation of interactive keys based on SDD-documents will greatly improve the user-friendliness of portal systems across platforms.

With this, the different platforms will for the first time be able to mutually benefit from their respective strengths. The new development will represent an important cornerstone for the establishment of a harmonised and consistent international biodiversity information infrastructure.
